# Hemoglobin A_1c_ Levels Predicts Acute Kidney Injury after Coronary Artery Bypass Surgery in Non-Diabetic Patients

**DOI:** 10.21470/1678-9741-2016-0010

**Published:** 2017

**Authors:** Cevdet Ugur Kocogulları, Atike Tekeli Kunt, Rezan Aksoy, Cagrı Duzyol, Hakan Parlar, Huseyin Saskın, Orhan Fındık

**Affiliations:** 1 Siyami Ersek Training and Research Hospital, Istanbul, Turkey.; 2 Derince Training and Research Hospital, Kocaeli, Turkey.; 3 Kartal Kosuyolu Training and Research Hospital, Istanbul, Turkey.

**Keywords:** Coronary Artery Bypass, Kidney, Dialysis, Acute Kidney Injury, Mammary Arteries

## Abstract

**Introduction:**

Elevated hemoglobin A_1c_ levels in patients with diabetes mellitus have been known as a risk factor for acute kidney injury after coronary artery bypass grafting. However, the relationship between hemoglobin A_1c_ levels in non-diabetics and acute kidney injury is under debate. We aimed to investigate the association of preoperative hemoglobin A_1c_ levels with acute kidney injury in non-diabetic patients undergoing isolated coronary artery bypass grafting.

**Methods:**

202 non-diabetic patients with normal renal function (serum creatinine <1.4 mg/dl) who underwent isolated coronary bypass were analyzed. Hemoglobin A_1c_ level was measured at the baseline examination. Patients were separated into two groups according to preoperative Hemoglobin A_1c_ level. Group 1 consisted of patients with preoperative HbA_1c_ levels of < 5.6% and Group 2 consisted of patients with preoperative HbA_1c_ levels of ≥ 5.6%. Acute kidney injury diagnosis was made by comparing baseline and postoperative serum creatinine to determine the presence of predefined significant change based on the Kidney Disease Improving Global Outcomes (KDIGO) definition.

**Results:**

Acute kidney injury occurred in 19 (10.5%) patients after surgery. The incidence of acute kidney injury was 3.6% in Group 1 and 16.7% in Group 2. Elevated baseline hemoglobin A_1c_ level was found to be associated with acute kidney injury (*P*=0.0001). None of the patients became hemodialysis dependent. The cut off value for acute kidney injury in our group of patients was 5.75%.

**Conclusion:**

Our findings suggest that, in non-diabetics, elevated preoperative hemoglobin A_1c_ level may be associated with acute kidney injury in patients undergoing coronary artery bypass grafting. Prospective randomized studies in larger groups are needed to confirm these results.

**Table:** 

Abbreviations, acronyms & symbols	
ACT	= Activated clotting time
AKI	= Acute kidney injury
AUC	= Area under curve
BUN	= Blood urea nitrogen
CABG	= Coronary artery bypass grafting
CPB	= Cardiopulmonary bypass
DM	= Diabetes mellitus
GFR	= Glomerular filtration rate
HbA1c	= Hemoglobin A1c
ICU	= Intensive care unit
KDIGO	= Kidney Disease Improving Global Outcomes
LAD	= Left anterior descending artery
LIMA	= Left internal mammary artery
LVEF	= Left ventricular ejection fraction
ROC	= Receiver operating characteristic

## INTRODUCTION

Coronary artery bypass grafting (CABG) operations are performed safely and successfully in our country as well as in the rest of the world. Acute kidney injury (AKI), not rarely seen following cardiac surgery, is associated with morbidity, increased health costs, and mortality rates^[1,2]^.

The risk factors and pathophysiology of AKI following CABG were described in the literature and have been the subject of multiple studies^[3,4]^.

The incidence of AKI following cardiac surgery has been reported as being 5-30% and renal replacement therapy is required in 1-2% of these patients^[5,6]^. Hemoglobin A_1c_ (HbA_1c_) is widely used as a marker of average blood glucose concentrations over the preceding 2 to 3 months and it has advantages over glucose tests^[7]^. Some evidence indicates that high HbA_1c_ levels prior to surgery are strongly associated with the severity of adverse events after CABG^[8]^.

HbA_1c_ levels were found to be related to cardiovascular and renal complications following open heart surgery^[9]^. Multiple factors have been implicated as contributors to postoperative AKI, including advanced age, female gender, presence of diabetes mellitus, chronic kidney disease, extended time between heart catheterization and surgery, aortic cross clamp time, duration of cardiopulmonary bypass (CPB), and blood transfusion following surgery^[6]^.

However, association of elevated HbA_1c_levels in non-diabetics with AKI after CABG surgery is under debate. The purpose of this study is to investigate the association of preoperative HbA_1c_ levels in non-diabetics with AKI after isolated CABG.

## METHODS

In this study, medical records of 315 open cardiac surgery patients operated in the same center by the same surgical team between June 2012 and July 2014 were investigated consecutively and retrospectively. Patients who underwent isolated CABG with CPB and who were non-diabetic with preoperative serum creatinine levels less than 1.4 mg/dl were included in the study. The number of patients that met that criteria was 202. For descriptive purposes, receiver operating characteristic (ROC) curve analysis was performed to identify the cut-off point with the highest sensitivity and specificity. Patients were grouped according to HbA_1c_ status: < 5.6% (low HbA_1c_ group; group 1) and ≥5.6% (high HbA_1c_ group; group 2).

AKI diagnosis was made by comparing baseline and postoperative serum creatinine to determine the presence of predefined significant change based on the Kidney Disease Improving Global Outcomes (KDIGO) definition (increase in serum creatinine by ≥0.3 mg/dl within 48 hours of surgery or increase in serum creatinine to ≥1.5 times baseline within 3 days of cardiac surgery)^[10]^. AKI diagnosis was based on the highest serum creatinine concentration measured during the first 3 days after surgery compared to the baseline serum creatinine concentration, defined as the last concentration measured before surgery. Urine output was not used to define AKI because it may be influenced by diuretics administered during anesthesia and CPB.

**Fig. 1:**
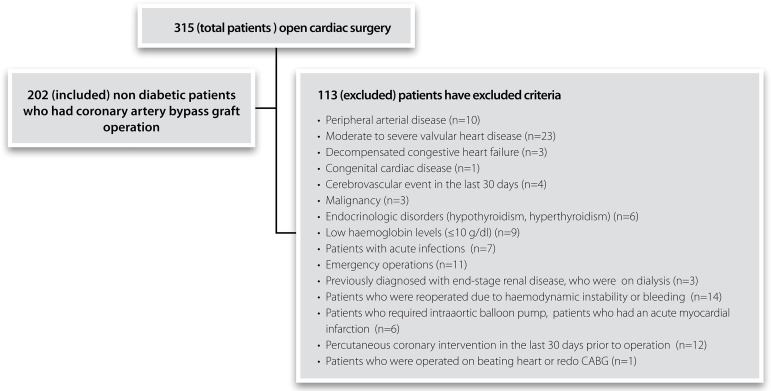
Consort diagram figure of patient selection.

Exclusion criteria included patients who had peripheral arterial disease, moderate to severe valvular heart disease, decompensated congestive heart failure, congenital cardiac disease, cerebrovascular event in the last 30 days, malignancy, endocrinological disorders (hypothyroidism, hyperthyroidism), low hemoglobin levels (≤10 g/dl), acute infections, emergency operations; patients who had previous diagnosis of end-stage renal disease and who were on dialysis; patients who were reoperated due to hemodynamic instability or bleeding; patients who required intra-aortic balloon pump; patients who had acute myocardial infarction and percutaneous coronary intervention in the last 30 days prior to operation; and patients who were operated on beating heart or redo CABG. A total of 113 patients were excluded from study, as shown in Figure 1. Additionally, patients for whom data on serum creatinine levels or urine output were missing were also excluded.

Patients' demographic and clinical data were obtained by using the hospital's software system of records and archives to investigate patient files, epicrisis, operation notes, and laboratory results. Age, gender, smoking history, hypertension, hyperlipidemia, left ventricular ejection fraction (LVEF), preoperative and postoperative laboratory parameters (hemoglobin, fasting blood glucose, creatinine, urea, creatinine clearance), perioperative data, duration of CPB and aortic cross clamp, amount of blood products used, and intensive care unit (ICU) and hospital length of stay were recorded.

Patients were followed in the ICU in the postoperative period, according to protocols of our institution. Electrocardiography, systemic mean arterial pressure, central venous pressure, arterial blood gases, chest tube drainage, and urine output were monitored. Preoperative and postoperative creatinine clearances and peak creatinine clearance were calculated according to the formulas reported in the literature^[11,12]^.

### Operative Technique

All of the patients were operated with median sternotomy under general anesthesia and CPB with aortic and venous cannulations following systemic heparin administration (300 IU/kg). Activated clotting time (ACT) was maintained at over 450 seconds during the operation. Standard CPB circuit and surgical management were used. Antegrade hypothermic and hyperkalemic blood cardioplegia was applied to all patients. Surgery was performed under moderate systemic hypothermia (28-30ºC). CPB flow was maintained at 2.2-2.5 l/min/m^2^, mean perfusion pressure was maintained between 50 and 80 mmHg, hematocrit level was maintained between 20 to 25% during CPB. For the coronary bypass operations, arterial grafts for left anterior descending artery (LAD) revascularization were preferably harvested from the left internal mammary artery (LIMA) whereas saphenous venous grafts were used for the other vessels. Distal anastomoses were done during aortic cross-clamp period and proximal anastomoses were done on beating heart onto the ascending aorta using a lateral clamp.

### Statistical Analysis

Statistical analysis was performed using SPSS version 13.0 (SPSS Inc, Chicago, IL, USA). Normal distribution was evaluated by histogram or Kolmogorov-Smirnov test; homogeneity of distribution was evaluated by 'Levene's test for equality of variance'. Normally distributed data were demonstrated as mean ± standard deviation whereas non-normally distributed data were demonstrated as median (minimum-maximum). Difference between groups was evaluated by 'Student's t test' in normal and homogenous distribution and by 'Mann-Whitney U test' in non-normal distribution. Differences between groups were evaluated by parametric or non-parametric Pearson Chi-Square test or Fisher's Exact test with respect to the distribution. Forward stepwise multivariate logistic regression models were created to identify the independent predictors of postoperative AKI. Variables with a *P* value less than 0.10 in univariate analyses were included in the multivariate model. The sensitivity and specificity of the independent risk factors to predict postoperative AKI were determined by ROC curve analysis. *P* value less than 0.05 was accepted as significant. Chi-square test was performed for odds ratio. Continuous variables were described as means (standard deviation) or medians (interquartile range), as appropriate; categorical variables were described as percentage.

## RESULTS

The demographic characteristics and clinical data of the patients were summarized in Table 1. There were no differences between the two groups in terms of demographic or clinical data.

**Table 1:** Demographic and clinical properties of the patients.

Characteristics	HbA_1c_ <5.6	HbA_1c_ ≥5.6	*P* value
	Group 1 (n=112)	Group 2 (n=90)	
Age, years Median (min-max)	63 (36-82)	60 (33-82)	0.47**
Male (%)	88 (78.6%)	74 (82.2%)	0.52*
Female (%)	24 (21.4%)	16 (17.8%)	0.52*
Hypertension (%)	73 (65.2%)	51 (56.7%)	0.22*
Hyperlipidemia (%)	50 (44.6%)	45 (50.0%)	0.45*
Smoking (%)	51 (45.5%)	33 (36.7%)	0.20*
Ejection fraction Median (min-max)	58 (25-70)	58 (30-70)	0.42**
EuroSCORE Median (min-max)	2 (0-6)	1 (0-6)	0.19**

* Pearson Chi-Square test or Fisher's Exact test;

** Mann-Whitney U test

Preoperative and postoperative blood analysis and haematological parameters of the patients were summarized in Table 2. First postoperative day creatinine levels (*P*=0.01), 3^rd^ postoperative day creatinine levels (*P*=0.0001), and 7^th^ postoperative day creatinine levels (*P*=0.0001) were significantly different between the groups.

**Table 2:** Preoperative and early postoperative blood results and haematological parameters of patient.

Preoperative and early postoperative blood results and haematological parameters	HbA_1c_ <5.6 Group 1 (n=112)	HbA_1c_ ≥5.6 Group 2 (n=90)	*P* value
	Median (min-max)	Median (min-max)	
Preoperative haemoglobin (mg/dl)	13.6 (10.4-16.5)	13.8 (10.5-16.0)	0.39**
Preoperative creatinine (mg/dl)	0.90 (0.60-1.38)	0.90 (0.60-1.30)	0.32**
Preoperative BUN (mg/dl)	16.0 (8.0-35.0)	17.0 (9.0-55.0)	0.10**
Preoperative creatinine clearance (ml/min)	109 (31-180)	111 (63-186)	0.96**
Preoperative fasting blood glucose (mg/dl)	94 (62-135)	95 (69-136)	0.74**
Postoperative first day hemoglobin (mg/dl)	9.1 (7.5-12.0)	9.0 (7.4-12.6)	0.13**
Postoperative first day creatinine (mg/L)	0.85 (0.50-2.00)	1.00 (0.56-2.60)	0.01**
Postoperative third day creatinine (mg/dl)	0.79 (0.40-1.70)	0.87 (0.50-3.90)	0.001**
Postoperative seventh day creatinine (mg/dl)	0.80 (0.50-1.80)	0.88 (0.50-2.80)	0.001**

BUN = blood urea nitrogen.

* Pearson Chi-Square test or Fisher's Exact test;

** Mann-Whitney U test

Intraoperative and postoperative data of the patients are shown in Table 3. ICU length of stay (*P*=0.004) was significantly different between the groups.

**Table 3:** Intraoperative and postoperative data of the patients.

Characteristics	HbA1c <5.6 Group 1 (n=112)	HbA1c ≥5.6 Group 2 (n=90)	*P* value
Aortic cross clamp time (minutes) Median (min-max)	60 (12-102)	57.5 (16-97)	0.94**
CPB time (minutes) Median (min-max)	95 (30-138)	93.5 (37-138)	0.75**
Use of blood products	48 (42.9%)	44 (48.9%)	0.39*
Use of inotropic support	20 (17.9%)	14 (15.6%)	0.66*
Amount of drainage (ml) Median (min-max)	350 (150-1100)	350 (150-1200)	0.51**
Intubation time (hours) Median (min-max)	5 (3-9)	5 (3-12)	0.74**
Intensive care unit stay (hours) Median (min-max)	21 (17-46)	21.5 (18-67)	0.004**

CPB = cardiopulmonary bypass.

* Pearson Chi-Square test or Fisher's Exact test;

** Mann-Whitney U test

Postoperative AKI occurred in 4 (3.6%) patients in group 1 and in 15 (16.7%) in group 2, showing a statistically significant difference between the groups (*P*=0.0002). Mortality in the early postoperative period occurred in 2 (1.8%) patients in group 1 and in 6 (6.7%) in group 2, and there was no statistically significant difference between the groups (*P*=0.14). Renal replacement therapy in the early postoperative period was required in 4 (3.6%) patients in group 1 and in 6 (6.7%) in group 2, showing no statistically significant difference between the groups (*P*=0.35).

Sensitivity and specificity of preoperative HbA_1c_ levels to predict AKI in non-diabetic patients after CABG was 79% and 59%, respectively. Positive predictive and negative predictive values were 17% and 96%, respectively. Preoperative HbA_1c_ levels higher than 5.6% had an odds ratio of 5.41 for AKI.

Results of univariate and multivariate regression analyses of preoperative risk factors that may influence the development of AKI after CABG in non-diabetic patients are shown in Table 4. In univariate regression analysis, preoperative creatinine (*P*=0.001), preoperative blood urea nitrogen (BUN) (*P*=0.0001), and preoperative HbA_1c_ (*P*=0.0001) levels were found to be associated with postoperative AKI occurrence. In multivariate regression analysis, HbA_1c_ (OR 11.17, 95% CI:2.21-56.33, *P*=0.003) was found to be independently associated with an increased risk for AKI.

**Table 4:** Univariate and multivariate regression analysis of preoperative risk factors for acute kidney injury.

	Acute Kidney Injury			
Variables	Unadjusted OR (95% CI)	*P*	Adjusted	*P*
			OR (95% CI)	
Male gender	0.66 (0.22-1.96)	0.46	-	-
Age	1.04 (0.99-1.09)	0.16	-	-
Ejection fraction	1.04 (0.98-1.11)	0.18	-	-
Hypertension	1.41 (0.51-3.87)	0.51	-	-
Hyperlipidaemia	1.28 (0.50-3.30)	0.61	-	-
Smoking	0.62 (0.23-1.71)	0.36	-	-
Preoperative fasting blood glucose	0.98 (0.95-1.01)	0.18	-	-
Preoperative creatinine	49.36 (4.69-519.63)	0.001	6.21 (0.27-144.64)	0.26
Preoperative hemoglobin	0.83 (0.60-1.14)	0.24	-	-
Preoperative BUN	1.15 (1.08-1.23)	0.0001	1.07 (0.98-1.17)	0.11
Preoperative creatinine clearance	0.99 (0.97-1.01)	0.22	-	-
Preoperative HbA1c	29.04 (6.49-130.03)	0.0001	11.17 (2.21-56.33)	0.003

BUN = blood urea nitrogen; HbA1c = Glycosylated Hemoglobin (Hemoglobin A_1c_)

**Fig. 2:**
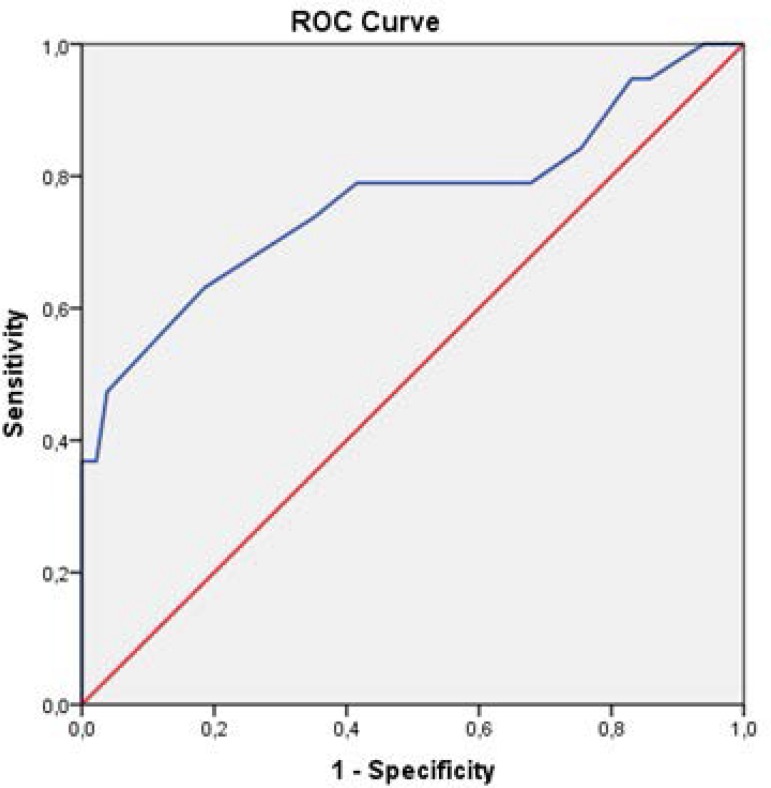
ROC curve analysis of preoperative HbA_1c_ regarding occurrence of postoperative AKI. Cut-off value for HbA_1c_ level was determined as 5.75%, at which sensitivity, specificity of the test, and AUC (area under curve) were calculated as 73.7%, 65.0%, and 0.76 (95% CI=0.62-0.95, P=0.0001), respectively

ROC curve analysis of HbA_1c_ level, which was found to be a risk factor for postoperative AKI occurrence in multivariate regression analysis, is depicted in Figure 2. Cut-off value for HbA_1c_ level was determined as 5.75%, at which sensitivity, specificity of the test, and AUC (area under curve) were calculated as 73.7%, 65%, and 0.76 (95% CI=0.62-0.95, *P*=0.0001), respectively.

### Study Limitations

There are some limitations to our study. This study was carried out at a single center, with a limited number of patients, and it was designed as a retrospective study rather than a randomized trial.

## DISCUSSION

AKI, not rarely seen following cardiac surgery, prolongs ICU and hospital length of stay and results in increased health costs and mortality rates^[1,2]^.

AKI following cardiac surgery is a multifactorial state. Risk factors are advanced age, presence of diabetes mellitus, hypertension, low preoperative glomerular filtration rate (GFR) (<60 ml/min/m^2^), left ventricular systolic dysfunction (LVEF<35%), preexisting kidney dysfunction, atherosclerosis of the ascending aorta, urgent or emergent surgery following myocardial infarction or percutaneous cardiac intervention, and administration of nephrotoxic agents^[13,14]^. Intraoperative factors also contribute to the development of AKI during cardiac surgery, such as renal hypoperfusion, nonpulsatile flow, and systemic inflammatory response syndrome due to CPB^[15,16]^. Demographic data and risk factors for AKI such as hypertension, low ejection fraction and EuroSCORE values were similar in both groups.

Long term survival of patients operated for cardiac surgery is directly proportional to the severity of AKI, which is related to changes in serum creatinine levels^[17]^. Our results showed that patients with higher preoperative HbA_1c_ levels had higher creatinine levels at 1^st^, 3^rd^ and 7^th^ postoperative days. Nevertheless, there was no difference in mortality. Instead, there was a significant difference when associated with prolonged ICU stay.

AKI following CPB is an important cause of morbidity and mortality^[18]^. Postoperative AKI requiring renal replacement has an independent effect on morbidity and early mortality. It is reported that the overall mortality due to AKI is 40-80%^[11]^. In the recent literature, there are several studies regarding early diagnosis of AKI and prevention of the inflammation process that is an accepted cause of AKI^[19,20]^. In a study by Freeland et al.^[6]^, blood transfusion was found as an independent risk factor for development of AKI following cardiac surgery. The same study also mentioned that longer aortic cross clamp and CPB times increased the incidence of AKI following cardiac surgery^[6]^. In a study by Khilji et al.^[21]^, both CPB and total cross-clamp times have been known as potential risk factors for developing kidney injury. In contrast with the literature, we did not find any significant differences between patients with AKI and without AKI regarding CPB and aortic cross clamp times and usage of blood products.

High mortality and morbidity rates following CABG operations have been reported in several studies in patients with type 2 diabetes mellitus (DM)^[22]^. In addition, some studies have shown that type 2 DM increases postoperative AKI after CABG^[23]^. HbA_1c_ level is a parameter used to evaluate long term glycemic control in patients with DM^[22]^. The American Diabetes Association included HbA_1c_ level in the criteria for diagnosing DM^[24]^. Normal HbA^1c^ levels are accepted as 4-6%. Tekumit et al.^[25]^ found that the borderline level of HbA_1c_ was 6.1% for patients undergoing CABG. In their retrospective study, Hudson et al.^[26]^ reported that preoperative HbA^1c^ levels over 6% were associated with 30-days postoperative mortality and occurrence of AKI in patients without DM who underwent open cardiac surgery.

In our study, patients were grouped according to HbA_1c_ levels, with borderline level being described as 5.6%. Patients with levels higher than 5.6% had significantly higher incidence of AKI, according to KGIDO classification. Our results revealed lower levels of HbA^1c^ than other studies as a risk factor for AKI^[27,28]^.

According to the KDIGO 2012 AKI Guideline, cardiac surgery with CPB is a 1B risk factor^[28]^. Despite the lack of consensus on AKI and HbA_1c_ levels in patients with no known renal disease, HbA^1c^ over 7% is defined as a Class 1A risk factor for patients with chronic renal disease^[28]^. The cut-off value for AKI in our group of patients was 5.75%.

Azevedo et al.^[29]^ observed that, in critical illness, there was a significant correlation between blood glucose levels and the incidence of AKI. Halkos et al.^[30]^ found that HbA^1c^ levels greater than 7% were associated with renal failure. Additionally, Gumus et al.^[9]^ found that elevated levels of HbA^1c^ were associated with increased renal complications. Likewise, in our study, a relationship was found between high preoperative creatinine, BUN, HbA_1c_ levels and occurrence of postoperative AKI in our study. It was also observed that average HbA_1c_ level in the preoperative period is a predictor of AKI in the early postoperative period following CABG.

## CONCLUSION

AKI following cardiac surgery causes multiple postoperative complications and leads to prolonged hospitalization, increased costs, and eventually increased mortality rates. Our results suggest that elevated preoperative HbA_1c_ level is associated with postoperative AKI and prolonged ICU stay in non-diabetic patients undergoing CABG. However, further prospective randomized studies are warranted to confirm these results.

**Table:** 

Authors' roles & responsibilities	
CUK	Conception and study design; execution of operations; analysis and/or data interpretation; statistical analysis; manuscript writing or critical review of its content; final manuscript approval
ATK	Conception and study design; execution of operations; analysis and/or data interpretation; statistical analysis; manuscript writing or critical review of its content; final manuscript approval
RA	Conception and study design; execution of operations; analysis and/or data interpretation; statistical analysis; manuscript writing or critical review of its content; final manuscript approval
CD	Conception and study design; execution of operations; analysis and/or data interpretation; statistical analysis; manuscript writing or critical review of its content; final manuscript approval
HP	Conception and study design; execution of operations; analysis and/or data interpretation; statistical analysis; manuscript writing or critical review of its content; final manuscript approval
HS	Conception and study design; execution of operations; analysis and/or data interpretation; statistical analysis; manuscript writing or critical review of its content; final manuscript approval
OF	Conception and study design; execution of operations; analysis and/or data interpretation; statistical analysis; manuscript writing or critical review of its content; final manuscript approval
